# Puerarin protects renal ischemia-reperfusion injury in rats through NLRP3/Caspase-1/GSDMD pathway

**DOI:** 10.1590/acb387323

**Published:** 2023-12-01

**Authors:** Kangyu Wang, Zhao Tang, Shuai Liu, Yan Liu, Huiqing Zhang, Haocheng Zhan

**Affiliations:** 1 The First Affiliated Hospital of Xinxiang Medical University – Department of Urology – Weihui – China.; 2 The First Affiliated Hospital of Xinxiang Medical University – Life Science Center – Weihui – China.

**Keywords:** Reperfusion Injury, Pyroptosis, NLR Family, Pyrin Domain-Containing 3 Protein, Caspase-1. Gasdermins

## Abstract

**Purpose:**

To observe the effect of puerarin on renal ischemia-reperfusion (I/R) injury in rats, and to explore its mechanism based on NLRP3/Caspase-1/GSDMD pathway.

**Methods:**

Twenty-one Sprague-Dawley rats were divided into three groups: sham-operated group (sham), model group (RIRI), and puerarin treatment group (RIRI + Pue). The model of acute renal I/R injury was established by cutting the right kidney and clamping the left renal pedicle for 45 min.

**Results:**

Renal function parameters were statistically significant in group comparisons. The renal tissue structure of rats in sham group was basically normal. Pathological changes were observed in the RIRI group. The renal pathological damage score and apoptosis rate in the RIRI group were higher than those in the sham group, and significantly lower in the RIRI + Pue group than in the RIRI group. Indicators of oxidative stress–superoxide dismutase, malondialdehyde, and glutathione peroxidase–were statistically significant in group comparisons. Compared with the sham group, the relative expressions of NLRP3, Caspase-1 and GSDMD proteins in the RIRI group were increased. Compared with the RIRI group, the RIRI + Pue group had significant reductions.

**Conclusions:**

Puerarin can inhibit the activation of NLRP3/Caspase-1/GSDMD pathway, inhibit inflammatory response and pyroptosis, and enhance the antioxidant capacity of kidney, thereby protecting renal I/R injury in rats.

## Introduction

Renal ischemia-reperfusion injury (RIRI) is one of the main causes of acute kidney injury, because of the lack of early diagnostic methods and treatment against the cause of specific, so the poor prognosis of Sars-CoV^1^. Its pathogenic mechanism is complex, involving a series of pathophysiological processes such as endoplasmic reticulum stress, reactive oxygen species aggregation, apoptosis, and inflammation^2^. Among them, inflammatory response is an important pathogenesis^3^. NLRP3 inflammatory corpuscle in regulating the important function of the kidney inflammation has been in various kidney diseases, including RIRI damage model confirmed^4^^,^ 5. Studies have shown that activation of NLRP3 inflammatory corpuscle after renal ischemia reperfusion induces interleukin (IL)-1β, and IL-18, such as the release of inflammatory factor, thus further aggravating RIRI^6^^,^ 7.

Puerain, a kind of isoflavone glycoside, is the main active ingredient extracted from *Pueraria lobata*, a commonly used Chinese herbal medicine. It has the characteristics of low toxicity and high safety. It has been reported puerarin has a lipid-lowering, anti-inflammatory, anti-oxidation, and anti-tumor activity. Besides, it is immune regulation, a kidney, nerves, and heart protector, and it removes oxygen free radicals and other pharmacological effects^8^^–^ 10. However, whether NLRP3 inflammasome is involved in the protective effect of puerarin against RIRI remains unclear and will be further explored in this study.

## Methods

### Experimental animals and reagents

Twenty-one specific-pathogen free Sprague-Dawley rats (8 weeks old, male, weighing 250–300 g) were purchased from Henan Laboratory Animal Center, license number: SCXK (Yu) 2021-0009. The rats were kept at room temperature of 26 ± 2°C, air humidity of 40–60%, 12 hours of day and night alternating light and dark, and free access to food and water. This experiment was approved by the Ethics Committee of the First Affiliated Hospital of Xinxiang Medical University.

Puerarin injection (CRC double crane and pharmaceutical co, LTD, specifications: 2 mL: 0.1 g, H20043489) was approved by the state. The detection kits of superoxide dismutase (SOD), malondialdehyde (MDA) and glutathione peroxidase (GSH-PX) were purchased from Nanjing Jian-Jian Bioengineering Institute. IL-1β enzyme-linked immunosorbent assay (ELISA) kit was purchased from Servicebio, China. IL-18 ELISA kit was purchased from Cloud-clone corp., China. IL-6 and tumor necrosis factor (TNF)-a ELISA assay kit was purchased from Jiangsu MEIMIAN Industrial Co., LTD. NLRP3 and GSDMD antibodies were purchased from Affinity Corporation. Caspase-1 antibody was purchased from Geneng Biological Corporation, and the secondary antibody HRP-goat anti-mouse was purchased from Shanghai Searcare Corporation.

### Group and the model established in this paper

All rats were randomly divided into three groups: sham operation group (sham group), model group (RIRI group), and puerarin treatment group (RIRI + Pue group), with seven rats in each group. Three days after adaptive feeding, preconditioning was given, and the rats in the RIRI + Pue group were given intraperitoneally (i.p.) puerarin (50 mg/kg)once a day for seven consecutive days. The sham group and RIRI group were given equal amounts of physiological saline at the same time.

According to Li et al.^11^, such as method of establishing rat RIRI model, in the last 30 minutes after the treatment, i.p. 3% pentobarbital sodium (50 mg/kg) intraperitoneal injection was given. After the success of the anesthesia for abdominal skin, the rats were fixed lie on their back on. The skin was cut about 6 cm along the midline of the abdomen, the muscular layer was cut, the skin was pulled open with a retractor, and the intestinal tube was moved to the left side to fully expose and free the right kidney. After ligating the renal pedicle with 2-0 surgical silk, the right kidney was removed, and the intestinal tube was moved to the right side.

**Figure 1:**
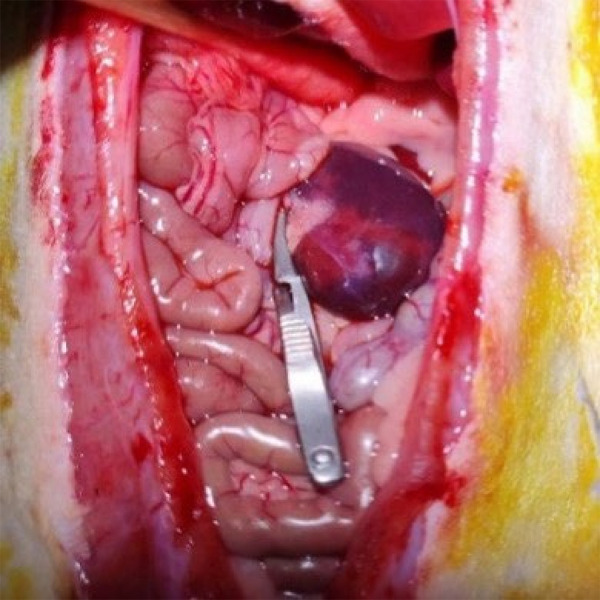
In ischemia.

**Figure 2:**
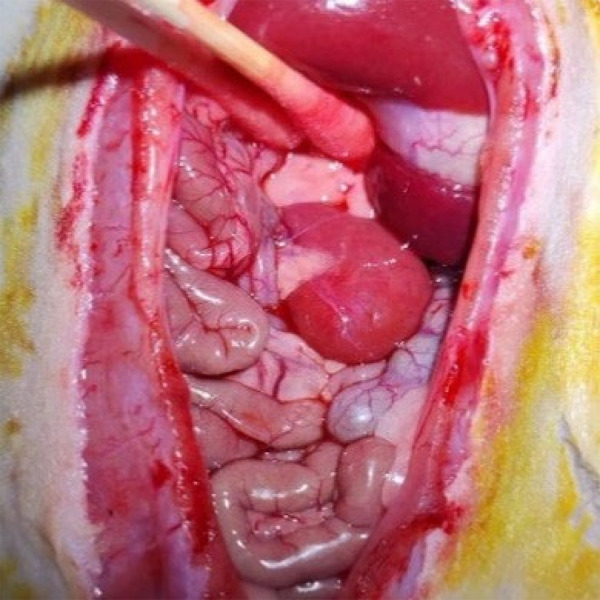
After ischemia.

With no damage after fully free the left renal pedicle arterial clip on the left side of the kidney, the visible color of the kidney changed from red to dark red, showing ischemia was successful (Fig. 1). It was removed after 45 min, and renal vascular clamp visible color from red to bright red showed that reperfusion was a success (Fig. 2). During ischemia, the intestine was kept warm by irradiation with a baking lamp and covered with warm saline gauze to prevent water evaporation. After observing that there was no bleeding in the abdominal cavity, the abdominal wall was sutured after the intestinal tube was correctly removed, and the incision was disinfected with iodine again. After the operation, the rats were irradiated with a baking lamp until they woke up. Sham group only free left renal pedicle, no clip, the rest of the same operation.

### Specimen acquisition

After 24 hours of reperfusion, the rats in each group were anesthetized, and 4-5-mL blood was collected through the abdominal aorta with a blood sampling needle in a biochemical coagulation tube. After standing at room temperature for 1 hour, the blood was centrifuged at 3,500 RPM for 10 minutes, and the upper layer of serum was collected. After blood collection, the left kidney was quickly dissociated and completely removed. Part of the left kidney was placed in 4% paraformaldehyde, and part was frozen at -80°C for subsequent detection of SOD, MDA, GSH-PX and protein extraction.

### Experimental methods

#### Determination of renal function

The levels of serum creatinine (SCr) and blood urea nitrogen (Bun) were detected by automatic biochemical analyzer (Shenzhen Radu Life Science Chemray 800).

#### Determination of serum inflammatory factors

Serum levels of IL-6 and TNF-α were determined by ELISA kit (MEIMIAN, JiangSu) according to the manufacturer’s instructions.

#### Pathological examination of renal tissue

Fresh renal tissues were fixed in 4% paraformaldehyde solution for 24 hours, embedded in paraffin, serially sectioned at 3 microns, and stained with hematoxylin and eosin. Then, five fields were randomly selected in the renal cortex under a light microscope (200x), five specimens in each group. The main observation indicators were tubular necrosis, cell vacuolization, tubular dilatation, tubular lumen cast formation, cell edema, inflammatory cell infiltration, etc. The degree of damage was determined according to the percentage of renal tubular damage^12^^,^ 13. The renal damage score was performed by two pathologists who were unaware of this experiment, and the scoring criteria were as follows: 1 (< 10%), 2 (10–25%), 3 (26–50%), 4 (51–75%), 5 (> 75%), as shown in Table 1 14.

**Table 1:** Renal tubular injury score.

Score	The degree of damage
0	Normal
1	Renal tubular injury < 10%
2	Renal tubular injury 10-25%
3	Renal tubular injury 26-50%
4	Renal tubular injury 51-75%
5	Renal tubular injury > 75%

Source: Adapted from Shan et al.^14^.

#### Apoptosis detection

DNA nucleotides terminal transferase mediated Nick-end labeling method (terminal-deoxynucleotidyl transferase mediated Nick end labeling–TUNEL) detection of renal tissue cell apoptosis was used.

#### Detection of IL-1β and IL-18 levels in renal tissue

Kidney tissues and ultrasonic grinding machine, extraction of protein, and protein concentration were measured by BCA kit after using IL-1β, and IL-18 assay kits. Both expression in kidney tissue was measured according to the instructions.

#### Determination of renal tissue oxidation markers

In 100-mg kidney tissue, according to the weight (mg), after centrifugation at 3,000 RPM for 10 minutes, the supernatant was taken for detection with SOD, MDA and GSH-PX kits (Nanjing Jianxian Bioengineering Institute).

#### Renal tissue inflammation related protein expression level detection

The protein expression levels of NLRP3, Caspase-1 and GSDMD in renal tissue were detected by Western blot. Tissue lysate (Biosharp BL504A) and Protease inhibitor (Servicebio G2007-1ML) were added to the frozen kidney tissue stored at -80°C, then placed in a grinding tube, and grinding beads were added. The addition ratio of kidney tissue and tissue lysate was 1:9.

After grinding, the supernatant was centrifuged at 12,000 RPM (4°C) for 5 minutes. The protein concentration was determined by the enhanced BCA protein assay kit. The protein standard curve was calculated to determine the loading amount, according to the protein concentration after the sample amount on sds-page electrophoresis.

### Statistical analysis

All data using Statistical Package for the Social Sciences (SPSS) and 25 GraphPad Prism 9.5 was analyzed, as well as the mean ± standard deviation (*x* + s), between multiple sets of the comparative analysis method for the single factor analysis of variance (ANOVA), and the comparative analysis between the two groups as *t* test. *P* < 0.05 for the difference was statistically significant.

## Results

### Effects of puerarin on renal function in rats

**Figure 3:**
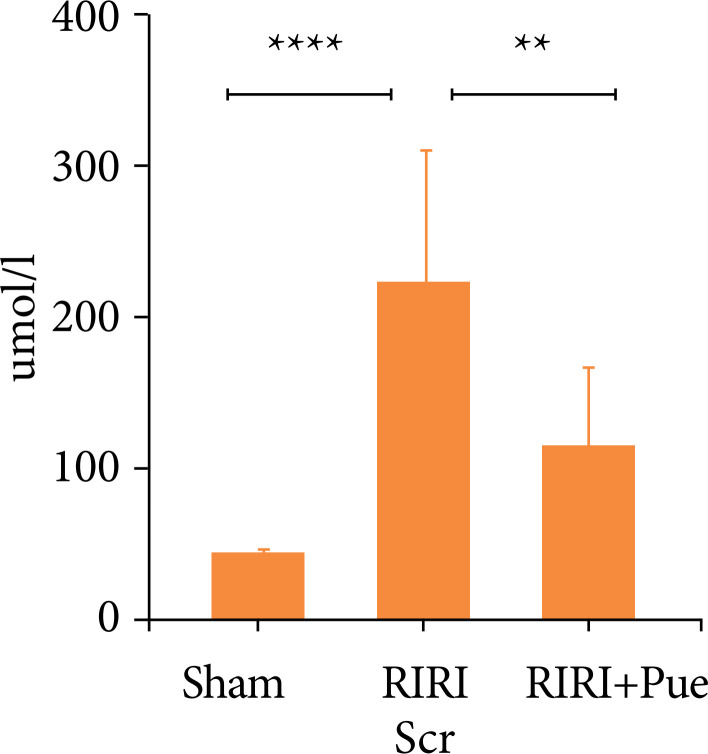
The levels of serum creatinine in three groups of rats.

RIRI: renal ischemia-reperfusion injury; Pue: puerarin; SCr: serum creatinine.

**Figure 4:**
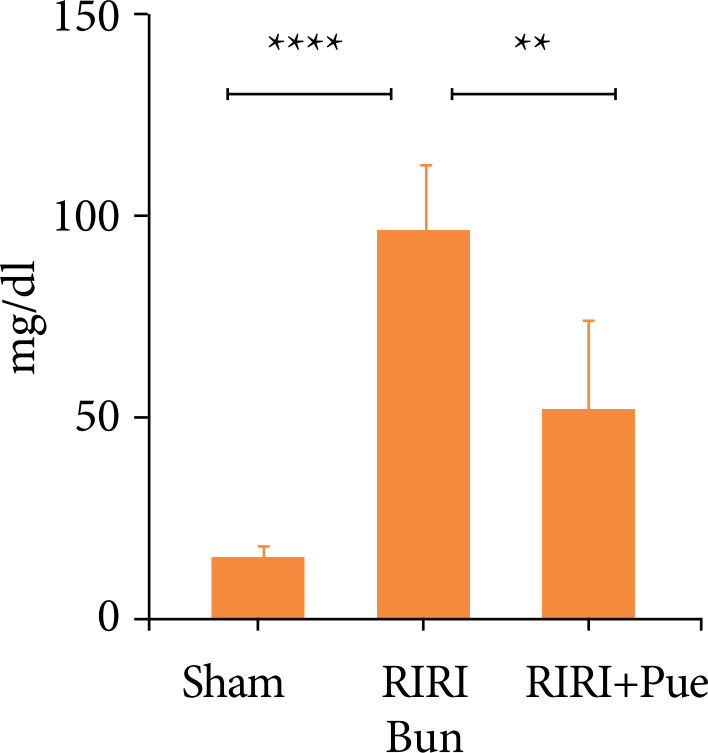
The levels of blood urea nitrogen in three groups of rats.

RIRI: renal ischemia-reperfusion injury; Pue: puerarin; Bun: blood urea nitrogen.

Compared with the control group (sham), in RIRI group, SCr, and Bun increased significantly (*P* < 0.05). Compared with the RIRI group, the RIRI + Pue group had relatively mild renal damage (*P* < 0.05). Puerarin has no damage to the kidney of rats and a certain protective effect on renal function damage caused by ischemia-reperfusion (Figs. 3 and 4).

### Puerarin in rat serum interleukin-6, the influence of tumor necrosis factor-α level

**Figure 5:**
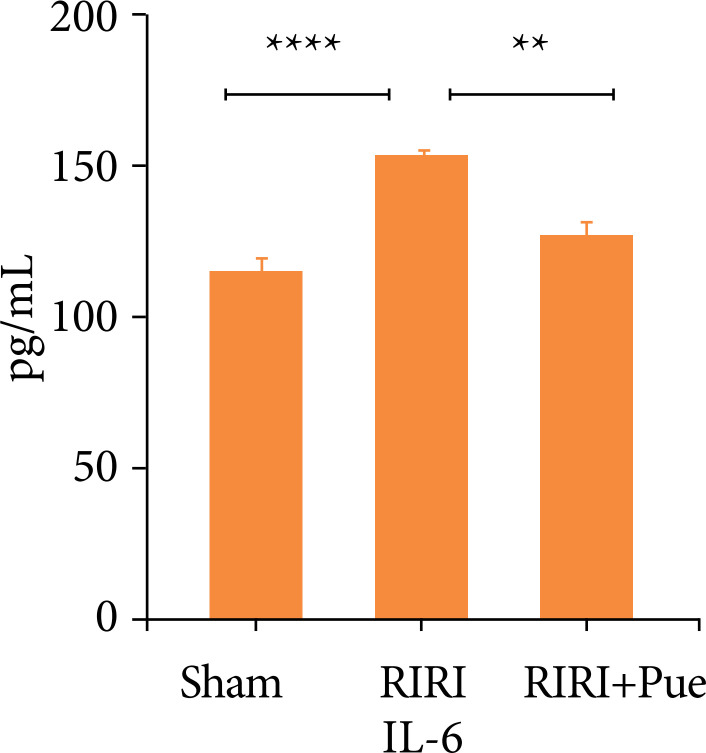
The levels of blood interleukin-6 in three groups of rats.

RIRI: renal ischemia-reperfusion injury; Pue: puerarin; IL: interleukin.

**Figure 6:**
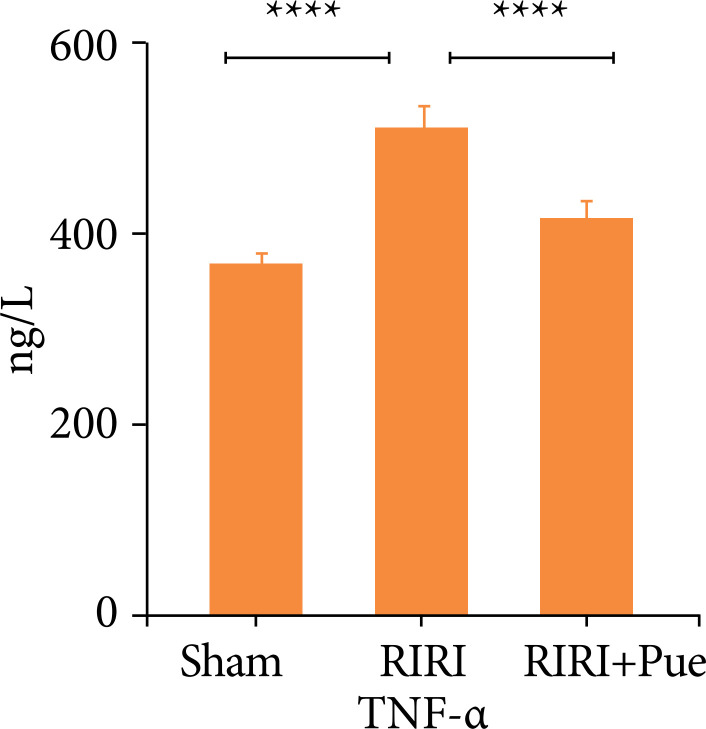
The levels of blood tumor necrosis factor-α in three groups of rats.

RIRI: renal ischemia-reperfusion injury; Pue: puerarin; TNF: tumor necrosis factor.

Compared with the sham operation group (sham), the expression levels of IL-6 and TNF-α in the serum of the RIRI group increased (*P* < 0.05). Compared with the RIRI group, the RIRI + Pue group had significant reductions in the expression of IL-6 and TNF-α (*P* < 0.05). This indicates that puerarin has anti-inflammatory and clearance of inflammatory mediators in the body (Figs. 5 and 6).

### Results of pathological examination

**Figure 7:**
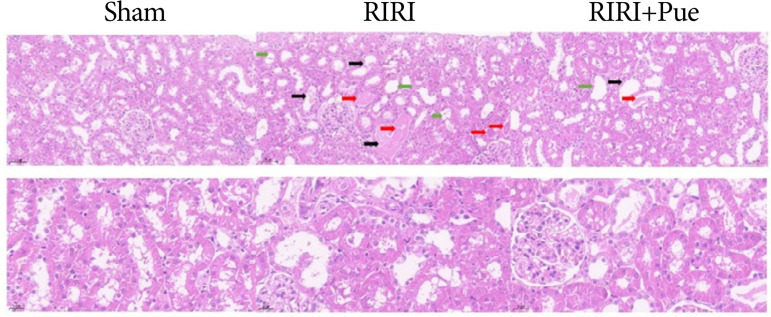
The pathological sections of kidneys were stained with hematoxylin and eosin in three groups of rats.

RIRI: renal ischemia-reperfusion injury; Pue: puerarin; *the upper is 200x magnification, the lowest is 400x magnification; black arrows indicate tubular dilatation; red arrows indicate protein casts; green arrows indicate renal tubular epithelial cells fall off. Source: elaborated by the authors.

**Figure 8:**
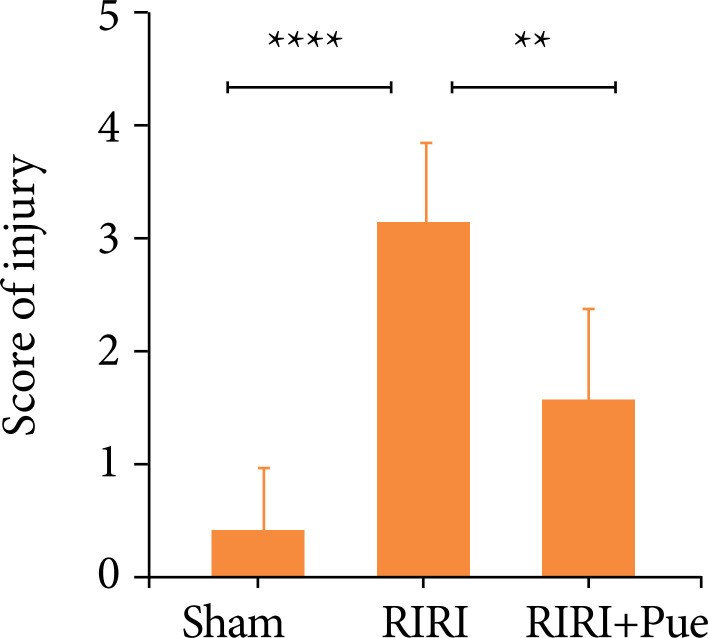
The scoring of pathological injuries of kidneys in three groups of rats.

RIRI: renal ischemia-reperfusion injury; Pue: puerarin.

In sham group, glomeruli and renal tubules were intact, renal tubular epithelial cells did not shed, and no abnormal pathological changes were observed. The RIRI group showed renal interstitial hemorrhage and inflammatory cell infiltration, varying degrees of cell edema, necrosis, cell vacuolar degeneration, and tubular cast formation (Fig. 7). The above pathological changes were significantly alleviated in the RIRI + Pue group (*P* < 0.05), as shown in Fig. 8. The results of hematoxylin and eosin staining showed that puerarin had a certain protective effect on tissue structure damage caused by RIRI.

### Detection results of puerarin on apoptosis

**Figure 9:**
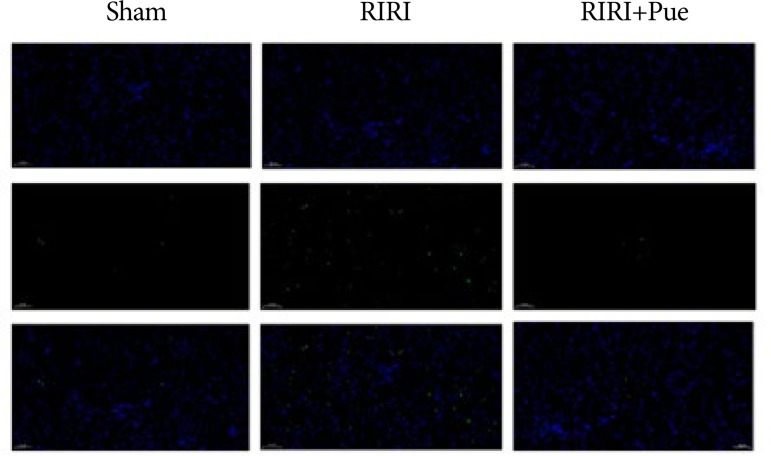
The fluorescence staining of pyroptosis of kidneys in three groups of rats.

RIRI: renal ischemia-reperfusion injury; Pue: puerarin.

**Figure 10:**
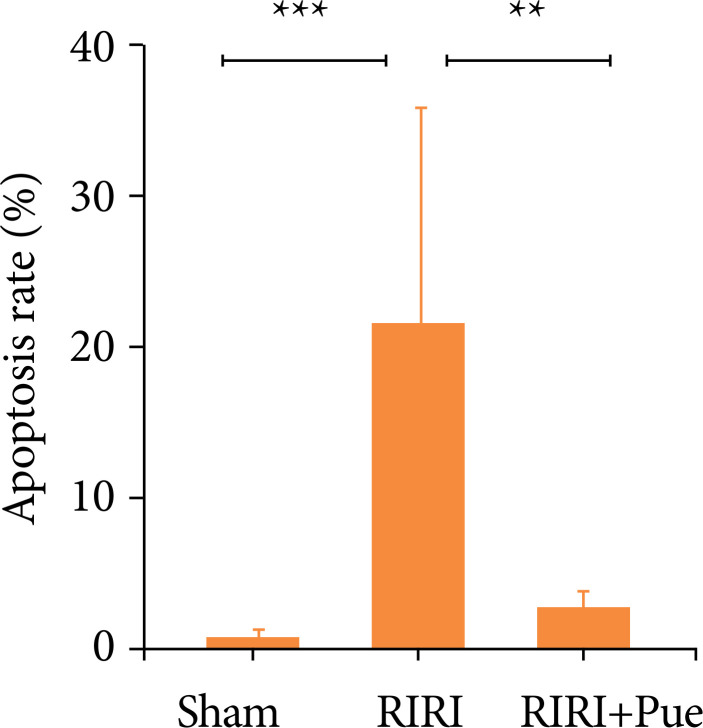
The ratio of pyroptosis of kidneys in three groups of rats.

RIRI: renal ischemia-reperfusion injury; Pue: puerarin.

Compared with the sham group, the RIRI group had a significantly increased rate of renal cell pyroptosis (*P* < 0.05), and compared with the RIRI group, the RIRI + Pue group had a significantly decreased rate of renal cell apoptosis (*P* < 0.05), indicating that puerarin could inhibit RIRI-induced pyroptosis (Figs. 9 and 10).

### Effect of puerarin on inflammatory factors in rat kidney tissue

**Figure 11:**
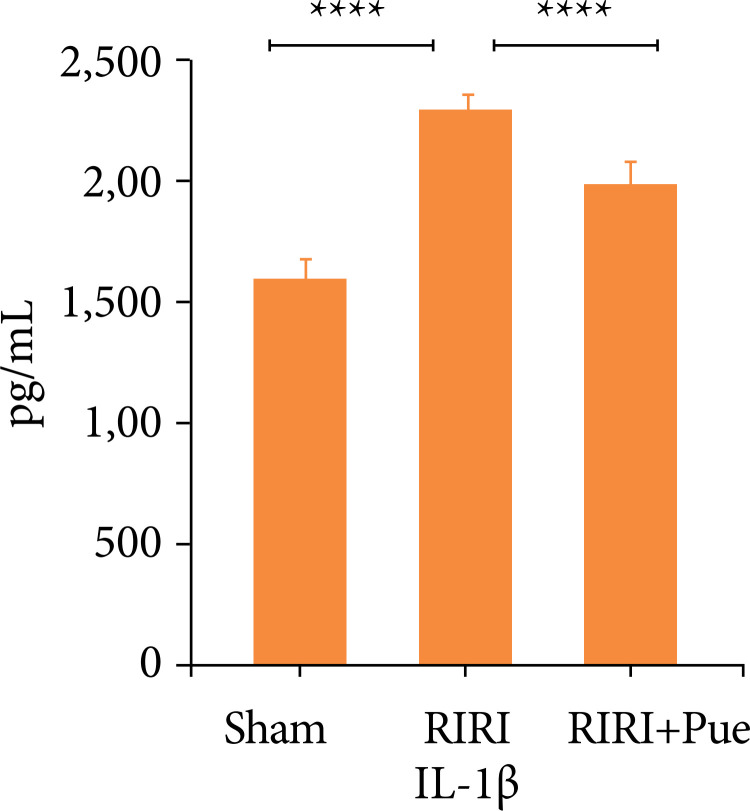
The levels of interleukin-1β of kidneys in three groups of rats.

RIRI: renal ischemia-reperfusion injury; Pue: puerarin; IL: interleukin.

**Figure 12:**
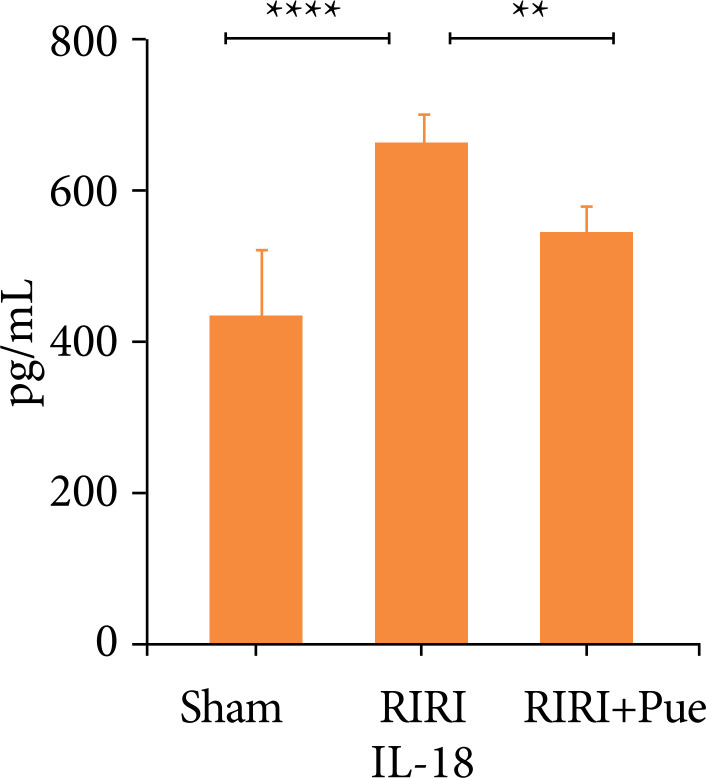
The levels of interleukin-18 of kidneys in three groups of rats.

RIRI: renal ischemia-reperfusion injury; Pue: puerarin; IL: interleukin.

Compared with the sham operation group, the expression levels of IL-1β and IL-18 in the renal tissue of the RIRI group were increased (*P* < 0.05). Compared with the RIRI group, the RIRI + Pue group had significant reductions in the expression of IL-1β and IL-18 (*P* < 0.05). This indicates that puerarin has anti-inflammatory and clearance of inflammatory mediators in the body (Figs. 11 and 12).

### Effects of puerarin on oxidative stress levels

**Figure 13:**
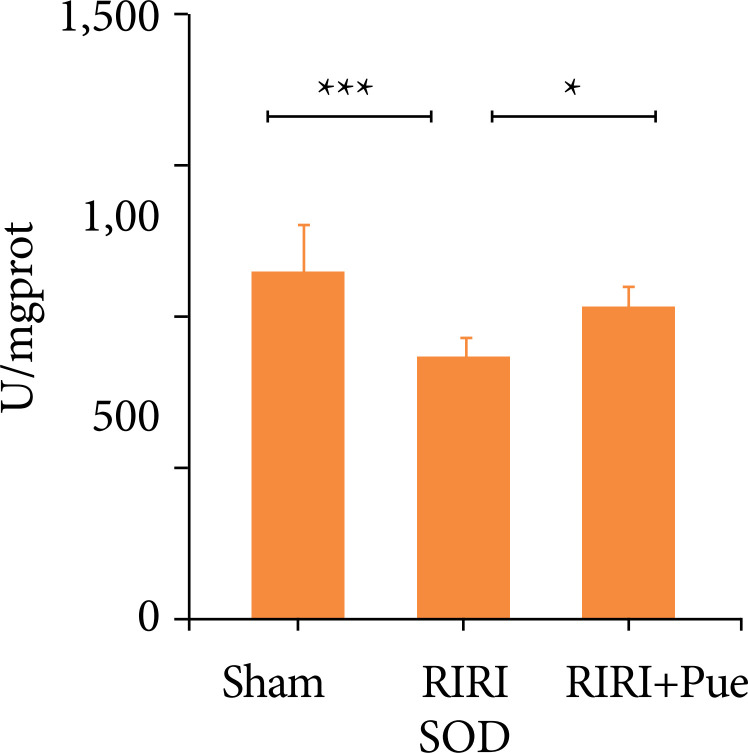
The levels of SOD of kidneys in three groups of rats.

RIRI: renal ischemia-reperfusion injury; Pue: puerarin; SOD: superoxide dismutase.

**Figure 14:**
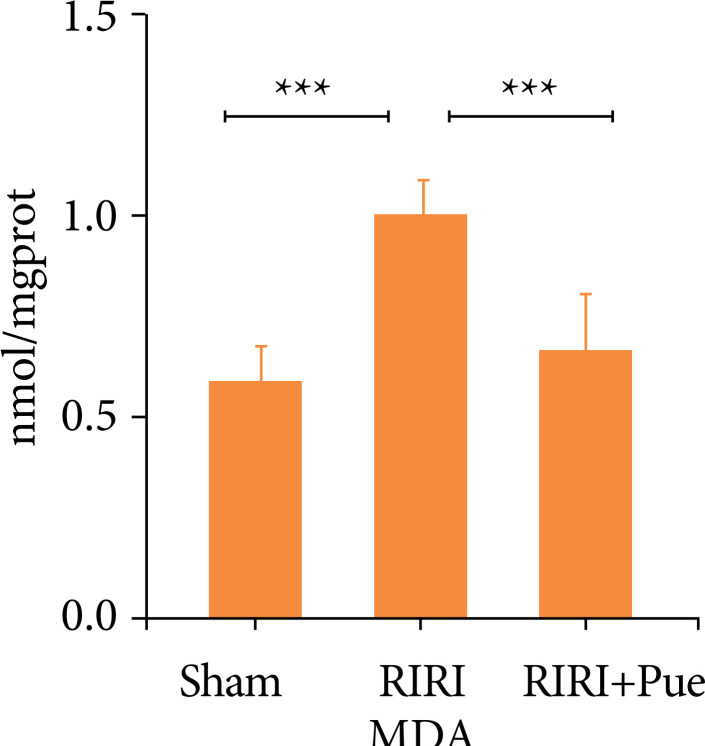
The levels of MDA of kidneys in three groups of rats.

RIRI: renal ischemia-reperfusion injury; Pue: puerarin; MDA: malondialdehyde.

**Figure 15:**
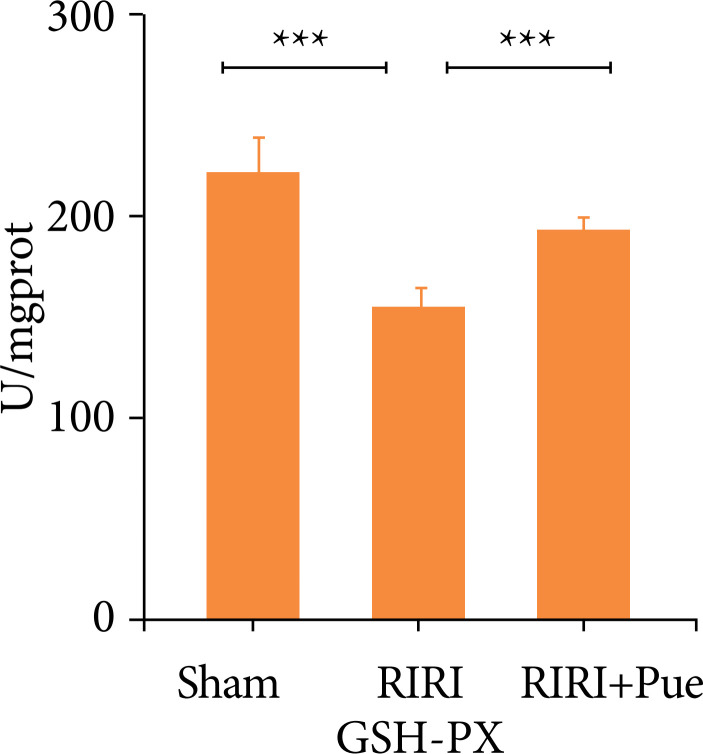
The levels of GSH-PX of kidneys in three groups of rats.

RIRI: renal ischemia-reperfusion injury; Pue: puerarin; GSH-PX: glutathione peroxidase.

Compared with the sham group, the levels of SOD and GSH-PX in the RIRI group were significantly decreased, and the level of MDA was significantly increased (*P* < 0.05). Compared with the RIRI group, the levels of SOD and GSH-PX in the RIRI + Pue group were significantly increased (*P* < 0.05), and the level of MDA was significantly decreased (*P* < 0.05). This suggests that puerarin is involved in the oxidative stress of RIRI and enhances the antioxidant activity of renal tissue (Figs. 13–15).

### Effect of puerarin on the expression of apoptosis-related proteins after renal ischemia-reperfusion injury in rats

**Figure 16:**
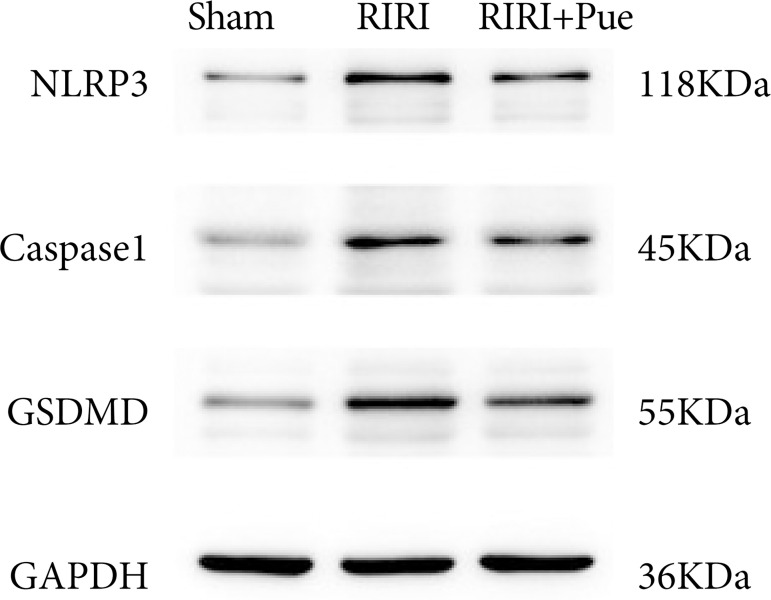
The western blotting of NLRP3, Caspase-1, and gasdermin D proteins in three groups of kidneys.

RIRI: renal ischemia-reperfusion injury; Pue: puerarin.

**Figure 17:**
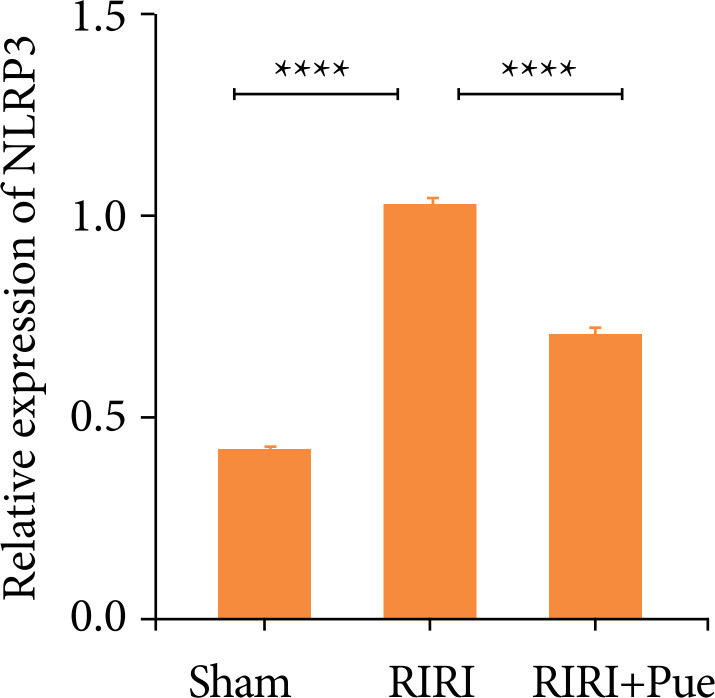
The levels of NLRP3 proteins in three groups of kidneys.

RIRI: renal ischemia-reperfusion injury; Pue: puerarin.

**Figure 18:**
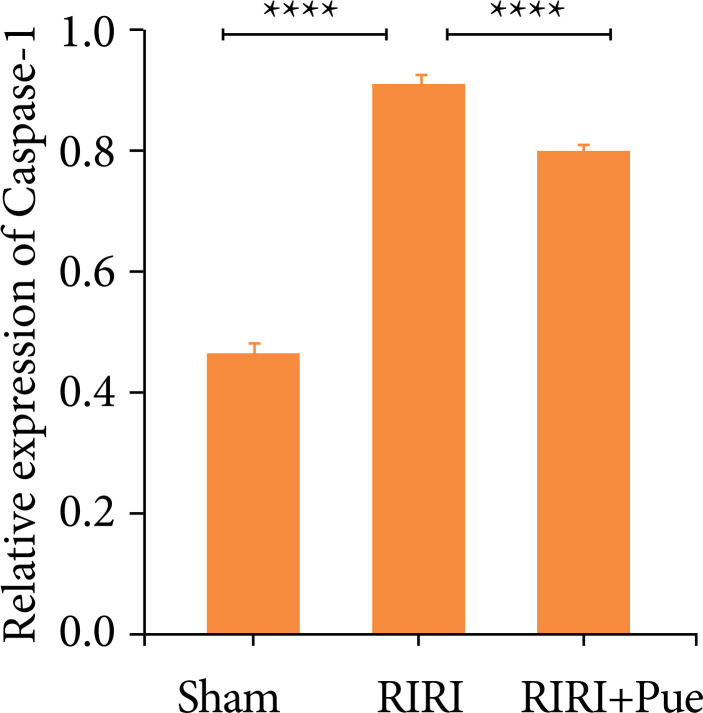
The levels of caspase-1 proteins in three groups of kidneys.

RIRI: renal ischemia-reperfusion injury; Pue: puerarin.

**Figure 19:**
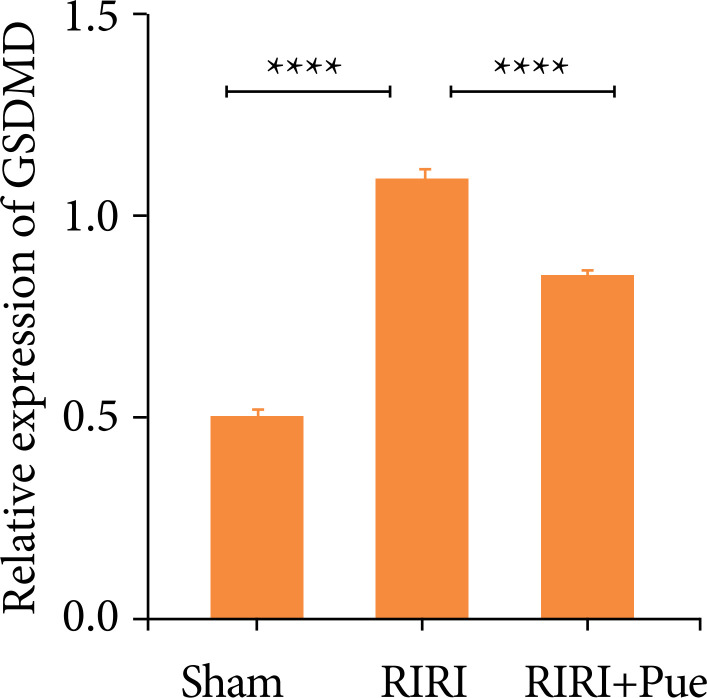
The levels of gasdermin D proteins in three groups of kidneys.

RIRI: renal ischemia-reperfusion injury; Pue: puerarin.

Compared with the sham group, the relative expression levels of NLRP3, Caspase-1, and GSDMD proteins in the RIRI group were increased (Fig. 16). Compared with the RIRI group, the RIRI + Pue group had significant reductions in the relative expression levels of NLRP3, Caspase-1 and GSDMD proteins (*P* < 0.05). The above results suggest that puerarin can inhibit the expression of these inflammatory proteins to vary degrees and attenuate acute kidney injury caused by inflammation (Figs. 17–19).

## Discussion

Acute kidney injury is a common acute disease with high mortality and poor prognosis, which is an increasing complication encountered in clinical practice^15^. RIRI is one of the important causes of acute kidney injury, which is common in renal transplantation, partial nephrectomy, shock, acute renal artery occlusion, and other urological surgeries^16^. Due to the complex pathophysiological mechanism of RIRI, there is no specific treatment nowadays. Therefore, elucidation of the underlying mechanisms and the search for effective treatments are urgent concerns.

Puerarin is a traditional Chinese medicine with a wide range of pharmacological effects and significant organ protection^17^. With the deepening of laboratory and clinical research on puerarin, it has been found that puerarin has a protective effect on ischemia-reperfusion injury of heart, brain, spinal cord, liver, intestine, and other organs^18^^–^ 20. Previous studies have shown that puerarin protects against myocardial IRI injury, cell damage caused by retinal IRI, and encephalopathy caused by brain IRI by inhibiting NLRP3 inflammasome^9^^,^ 21 ^,^ 22.

At the same time, Yu et al.^23^ showed that Pue had a protective effect on autophagy of hepatocytes by inhibiting NLRP3, Cai et al.^24^ showed that Pue had a protective effect on acute lung injury by regulating NLRP3-induced pyroptosis, Peng and Liu^25^ demonstrated puerarin has a protective effect on inflammatory injury of gastric epithelial cells by inhibiting apoptosis caused by NLRP3 inflammasomes^25^. However, whether puerarin exerts a protective effect on RIRI by inhibiting NLRP3 was investigated for the first time in this study.

In this study, the right kidney was removed, and the left kidney was clipped for 45 minutes to establish the model of acute RIRI. This method has been proved to be the best to ensure the survival rate of rats under the premise of successful RIRI model, and it can cause a certain degree of renal damage^26^^,^ 27. To observe the preventive effect of puerarin on RIRI, the rats were i.p. injected with a concentration of 50 mg/kg of puerarin seven days before modeling, and the rats were induced 30 minutes after the last administration. The results of the preliminary experiments showed that the serum levels of IL-6 and TNF-α in rats were at high levels after one day of reperfusion.

In addition, Choi et al. demonstrated that SCr and Bun reached the highest level 24 hours after RIRI, and began to decrease 48 hours later^28^. In this study, the rats were sacrificed one day after reperfusion, and blood and kidney samples were collected to evaluate the degree of renal damage and the level of inflammatory response. The results of this study showed that the serum levels of SCr, Bun, IL-6 and TNF-α in the RIRI group were significantly higher than those in the sham group. The pathological sections of hematoxylin and eosin staining showed renal interstitial hemorrhage, glomerular hypertrophy and inflammatory cell infiltration, varying degrees of cell edema and necrosis, and renal tubular tube formation. However, puerarin administration (RIRI + Pue group) significantly improved renal function, serum inflammation level and renal pathological structure. This suggests that puerarin can reduce renal pathological damage, protect renal function, and reduce serum inflammation levels, which is consistent with the previous research results of Jian et al.^29^.

Pyroptosis is regarded as a new inflammatory programmed cell death pathway, which has the characteristics of both apoptosis and necrosis, and is manifested as cell membrane rupture and cell content release, cell swelling and TUNEL staining positive, chromatin condensation, and DNA double-strand deletion^30^^,^ 31. Similarly, in the present study, the renal cell apoptosis rate was found to be significantly higher in the RIRI group than in the sham group, while it was significantly lower in the RIRI + Pue group, suggesting that preconditioning with puerarin could attenuate renal cell apoptosis.

It has been shown that the activation of NOD-like receptor family pyrin domain-containing 3(NLRP3) inflammasome is required for pyroptosis in various diseases^32^. NLRP3 inflammasome is a multimeric protein complex formed by the innate immune sensing protein NLRP3. After activation, NLRP3 binds to the adaptor protein ASC and recruits Caspase-1 to form NLRP3 inflammasome^33^. The assembly of NLRP3 inflammasome leads to the cleavage and activation of Caspase-1, further cleavage of gasdermin D (GSDMD), and the release of the N-terminal fragment of cleaved GSDMD (GSDMD-N), which binds to the phospholipid bilayer of the cell membrane, leading to the formation of membrane pores on the cell membrane, leading to cell swelling and membrane rupture^34^.

At the same time, activated Caspase-1 promotes the maturation and secretion of proinflammatory cytokines, such as IL-1β and IL-18^31^. Li et al.^35^ proved that the increased expression of NLRP3, Caspase-1, GSDMD and other marker proteins is an important feature that causes pyroptosis.

The results of this experiment showed that, compared with the sham group, the contents of IL-1β and IL-18 in renal tissue of the RIRI group were significantly increased, and the Western blotting results also showed that the expression levels of NLRP3, Caspase-1 and GSDMD proteins in renal tissue were significantly increased. Compared with the RIRI group, the RIRI + Pue group had significant reductions in the expression of IL-1β, IL-18, NLRP3, Caspase-1, and GSDMD proteins in renal tissues. The results of the present study suggest that puerarin attenuates RIRI by inhibiting the activation of NLRP3 inflammation and inhibiting the expression of markers such as Caspase-1 and GSDMD, as well as the release of inflammatory factors such as IL-1β and IL-18.

In the process of renal ischemia-reperfusion, excessive production of reactive oxygen species can cause renal tubular damage, endothelial dysfunction, and interstitial inflammation, so oxidative stress also plays a key role in the occurrence and development of RIRI^36^^,^ 37.

Huang et al.^38^ showed that puerarin, as an antioxidant, can maintain the activity of antioxidant enzymes and protect cells from apoptosis caused by oxidative stress. As a major antioxidant enzyme, SOD plays a crucial role in scavenging oxygen free radicals, and its activity can reflect the ability of the kidney to scavenge oxygen free radicals and resist lipid peroxidation. MDA content can reflect the content of oxygen free radicals, the degree of lipid peroxidation, and the degree of oxygen free radical damage to renal tissue. GSH-PX, an important enzyme that catalyzes the decomposition of peroxides, is an important scavenger of reactive oxygen species in the body.

This study also found that RIRI significantly increased MDA and decreased SOD and GSH-PX in the kidney of rats, while puerarin pretreatment downregulated MDA and upregulated SOD and GSH-PX levels, which were consistent with the results reported. Therefore, the present study suggests that the antioxidant effect of puerarin is one of the mechanisms to protect against RIRI.

## Conclusion

Puerarin can protect renal function, reduce the expression level of serum inflammatory mediators, protect renal pathological structure, and reduce renal tissue pyroptosis in the RIRI model. These protective effects were related to puerarin inhibiting the activation of NLRP3/Caspase-1/GSDMD pathway and preventing the initiation of pyroptosis pathway. This study elucidated part of the mechanism of RIRI, and puerarin is expected to be a candidate drug for clinical treatment of RIRI-related acute kidney injury. However, our study still has some limitations, and the specific mechanisms involved in RIRI are still unclear, which need to be further studied.

## Data Availability

All data sets were generated or analyzed in the current study.
